# Differences in Spatial Memory Recognition Due to Cognitive Style

**DOI:** 10.3389/fphar.2017.00550

**Published:** 2017-08-23

**Authors:** Laura Tascón, Maddalena Boccia, Laura Piccardi, José M. Cimadevilla

**Affiliations:** ^1^Department of Psychology, University of Almeria Almeria, Spain; ^2^Department of Psychology, ‘Sapienza’ University of Rome Rome, Italy; ^3^Cognitive and Motor Rehabilitation Unit, IRCCS Fondazione Santa Lucia Rome, Italy; ^4^Department of Life, Health and Environmental Sciences, L'Aquila University L'Aquila, Italy

**Keywords:** spatial memory, field dependence/independence, virtual reality, embedded figures test, environmental complexity

## Abstract

Field independence refers to the ability to perceive details from the surrounding context as a whole and to represent the environment by relying on an internal reference frame. Conversely, field dependence individuals tend to focus their attention on single environmental features analysing them individually. This cognitive style affects several visuo-spatial abilities including spatial memory. This study assesses both the effect of field independence and field dependence on performance displayed on virtual environments of different complexity. Forty young healthy individuals took part in this study. Participants performed the Embedded Figures Test for field independence or dependence assessment and a new spatial memory recognition test. The spatial memory recognition test demanded to memorize a green box location in a virtual room picture. Thereafter, during ten trials participants had to decide if a green box was located in the same position as in the sample picture. Five of the pictures were correct. The information available in the virtual room was manipulated. Hence, two different experimental conditions were tested: a virtual room containing all landmarks and a virtual room with only two cues. Accuracy and reaction time were registered. Analyses demonstrated that higher field independent individuals were related to better spatial memory performance in two landmarks condition and were faster in all landmark condition. In addition, men and women did not differ in their performance. These results suggested that cognitive style affects spatial memory performance and this phenomenon is modulated by environment complexity. This does not affect accuracy but time spent. Moreover, field dependent individuals are unable to organize the navigational field by relying on internal reference frames when few landmarks are available, and this causes them to commit more errors.

## Introduction

Spatial memory is a cognitive ability that permits the recollection of information about the space, its layout and locations (Castree et al., 2013). Spatial information can be analysed differently and allows diverse possibilities of action in a given spatial task (Kyritsis et al., 2009; Li et al., 2016). The distinct preferences to perceive and organize the information about the surrounding space are known as cognitive style (CS) (Hayes and Allinson, 1998; Smith and Riding, 2004; Kyritsis et al., 2009). Two opposite CS can be found: on the one hand, field independent (FI) participants can manage a holistic environmental representation, while, at the same time, they can perceive parts as a whole. On the other hand, field dependent (FD) subjects focus their attention on single environmental features by analyzing them individually (Witkin, 1977; Kyritsis et al., 2009).

The Embedded Figures Test is a paper-and-pencil task used to define the CS (Witkin et al., 1971). Participants are requested to search for a simple figure hidden in a complex one. Usually, FD individuals take longer to perform the task.

It is worthy to note that CS can predict performance on different spatial tasks. For instance, FI people are good at object rotation, perspective taking and using no-rotating maps (Boccia et al., 2016; Li et al., 2016). They have also been reported to handle more complex and flexible environmental representations (i.e., map-like representation) as compared with FD (Boccia et al., 2017).

Moreover, gender differences have been found in spatial tasks (Coluccia and Louse, 2004; Iachini et al., 2005; Cimadevilla et al., 2011; Piccardi et al., 2011a,b; Nori et al., 2015; León et al., 2016; Palmiero et al., 2016; Tascón et al., 2016, 2017). It is important to highlight that dimorphism depends on the task difficulty level, disappearing with low and very high demands (Coluccia and Louse, 2004; Nori and Piccardi, 2011; León et al., 2014; Tascón et al., 2017).

In addition to this, men and women are prone to use different strategies and spatial information to solve the same tasks (Coluccia and Louse, 2004; Driscoll et al., 2005; Woolley et al., 2010; Nori et al., 2015). In accordance with the Siegel and White's Model (see Siegel and White, 1975), the spatial CS corresponds to the type of information individuals select to navigate and orientate themselves in the environment. Generally speaking, women normally adopt a “landmark style” so as to “beacon” towards a salient landmark, using a sort of figurative memory, or a “route style” to navigate relying on the memory of the paths that connect different landmarks. Both styles are related to the use of egocentric strategies. Unlike them, men prefer to use “survey style,” a global map-like environmental representation associated with the use of allocentric strategies (Pazzaglia and De Beni, 2001; Coluccia and Louse, 2004; Nori et al., 2006). Considering field dependence/independence continuum, men have been reported to be FI while women are FD (Boccia et al., 2016).

On the other hand, spatial abilities have been assessed in humans using different methods. Virtual reality-based tasks have proved to be more accurate and useful to detect brain damages than classical neuropsychological tests (Cimadevilla et al., 2014). Indeed, spatial orientation in virtual environments is considered realistic enough to activate the same mechanisms involved during navigation in real environments both at behavioural and at neural levels (Aguirre et al., 1996).

Recently, a new spatial task was developed for assessing spatial memory in humans (Tascón et al., 2017). It demands participants to remember locations in a spatial recognition test and it has been reported as a good gender discrimination (Tascón et al., 2017).

The aim of the present study was to determine the effect of CS and gender on the performance in the spatial recognition task. According to previous literature, FI are better in handling spatial information, like perspective taking, a kind of process involved in the spatial recognition task used in this work. In addition, FI are more capable than FD in extracting important environmental information required for an accurate orientation. Taking into account that women are more often FD than men and two contexts with a different amount of landmarks will be used, we hypothesize that FI will show a better performance in spatial recognition than FD and we also expect that women will do better when all landmarks are available.

## Materials and methods

### Participants

The sample was made up of 40 undergraduate College students from “Sapienza” University, Rome (Italy). Twenty of them were men (Mean age = 25.7; *SD* = 2.8) and the other half women (Mean age = 25.7; *SD* = 2.3). None of them had a history of neurological or psychiatric diseases, which was later confirmed during an informal interview carried out before the test phase. In addition, all participants performed the Familiarity and Spatial Cognitive Style scale (FSCS; Piccardi et al., 2011c) which included 22 self-referential statements about various aspects of environmental spatial orientation. All participants self-classified themselves as having a “good or quite good sense-of-direction,” as evaluated by filling in the FSCS. Indeed, this scale was used to ensure that participants did not suffer from topographical orientation disorders. None of the participants showed the presence of navigational deficits or developmental topographical disorientation (see Iaria et al., 2005, 2009; Bianchini et al., 2010).

For demographic details see Table 1.

**Table 1:** Distribution of the sample in gender and age.

Males	*FI* = 11 *FD* = 9 Total = 20	*N* = 40 Mean age = 25.7 *SD* = 2.5
Females	*FI* = 9 *FD* = 11 Total = 20	

The study was developed under the European Community Council Directive, 2001/20/EC for biomedical research in humans. All subjects gave written informed consent.

### Instruments and procedure

The individual's predisposition toward the FD or the FI (i.e., CS) is usually assessed by tasks requiring participants to detect embedded simple figures in complex configurations (e.g., Witkin et al., 1971; Ekstrom et al., 1976). In these tasks, FI individuals, by ignoring contextual information, are better at detecting the embedded figures than FD individuals, who are more affected by the contextual information of the complex configurations and are less able at detecting the embedded figures in the whole configurations (Witkin, 1977; Witkin et al., 1977; Walter and Dassonville, 2011).

In the present study, we adopted the Embedded Figures Test (EFT) for assessing the participants' CS. The EFT is a paper-and-pencil test developed by Witkin et al. (1971) to analyse how an individual perceives and processes the surrounding field. It consists of a collection of cards 12.9 × 7.7 cm with complex and simple figures. Those simple figures are uncoloured and are formed by a single line. Complex figures are composed of a conjunction of small simple and multi-coloured figures. Each simple figure is hidden in the complex one. That is, the contour of the simple figure is formed by several substructures of the complex, so the simple one cannot be easily identified.

Each trial started showing a complex figure for 15 s. During this time participants had to describe it out in loud voice. Thereafter, the card with the simple figure overlapped the complex one for 10 s. After that, the experimenter removed the card with the simple figure and the participants had to find the contour of it inside the complex. They were instructed to inform the experimenter as soon as they found the simple figure and then to trace it by using a stylus. When a participant believed to have found the simple figure, the experimenter annotated the elapsed time (timing): if the response was correct, that time represented the response time; otherwise, if the response was wrong, the experimenter continued to clock the time until the participant reported the correct response or until 180 s had passed. The total time was computed by summing up the response time on each item, the result being divided by the number of items (Piccardi et al., 2011a) in order to compute the overall time averaged across items. Averaged times (EFT scores) were used as the measure of the individual's CS, with lower times indicating individuals with higher predisposition towards the FI.

As a scale for dividing subjects into FI and FD does not exist, and taking into account that the individual's predisposition to be field in/dependent is along a continuum, we decided to sort participants according to the median of the averaged times on the EFT. In such a way we divided participants into two groups: FI (i.e. higher times than median, that is faster individuals in detecting embedded figures) and FD (i.e. lower times than median, that is slower individuals in solving the EFT) groups.

The spatial recognition test (Tascón et al., 2017) was displayed on a Hewlett Packard 2600-MHz notebook equipped with 3 GB of RAM and a 15.4 Thin Film Transistor (TFT) colour screen (1920 × 1200 pixels). The recognition test was implemented in MATLAB using Cogent 2000 (Well- come Laboratory of Neurobiology, UCL, London, www.vislab.ucl.ac.uk/cogent.php).

**Figure 1:**
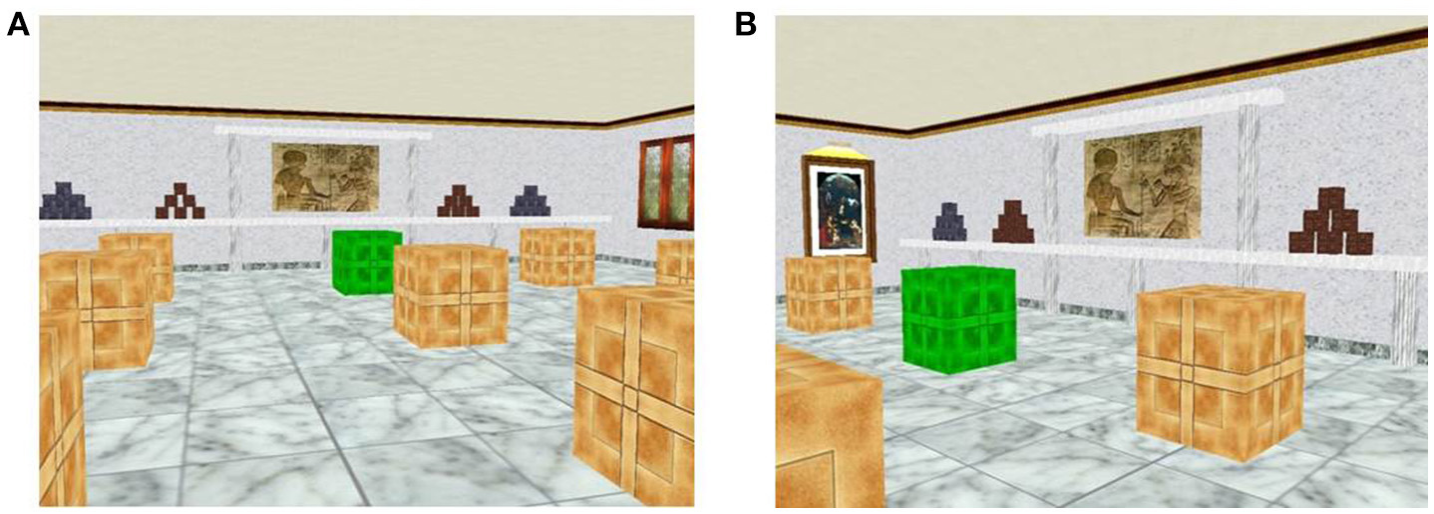
Two pictures from the spatial recognition task. **(A)** Learning phase: participants have to memorize the location of the green box. **(B)** Recognition phase: individuals have to indicate if the green box is in the same location than in the learning phase. Note that ten pictures were used in the recognition phase.

The spatial recognition task was based on the Almeria Spatial Memory Recognition Test (ASMRT) (Tascón et al., 2017). Instructions along with an example were displayed on the screen. The spatial task included two phases: learning and recognition phase. In the learning phase a picture showed a square virtual room with 9 boxes (3 × 3), one of them in green color. Participants were asked to memorize the green box location. No time limits were set. The recognition phase started when the space-key was pressed. A total of ten pictures in the virtual room was shown one by one. The virtual room was shown again with 9 boxes, one of them in green color. Participants had to decide if the green box corresponded spatially to the one of the sample phase. They had to provide positive or negative responses (yes/no) by pressing two buttons on the keyboard. The viewpoint changed across the pictures (see Figure 1). Five pictures represented correct locations. Both accuracy and reaction time were automatically recorded.

**Figure 2:**
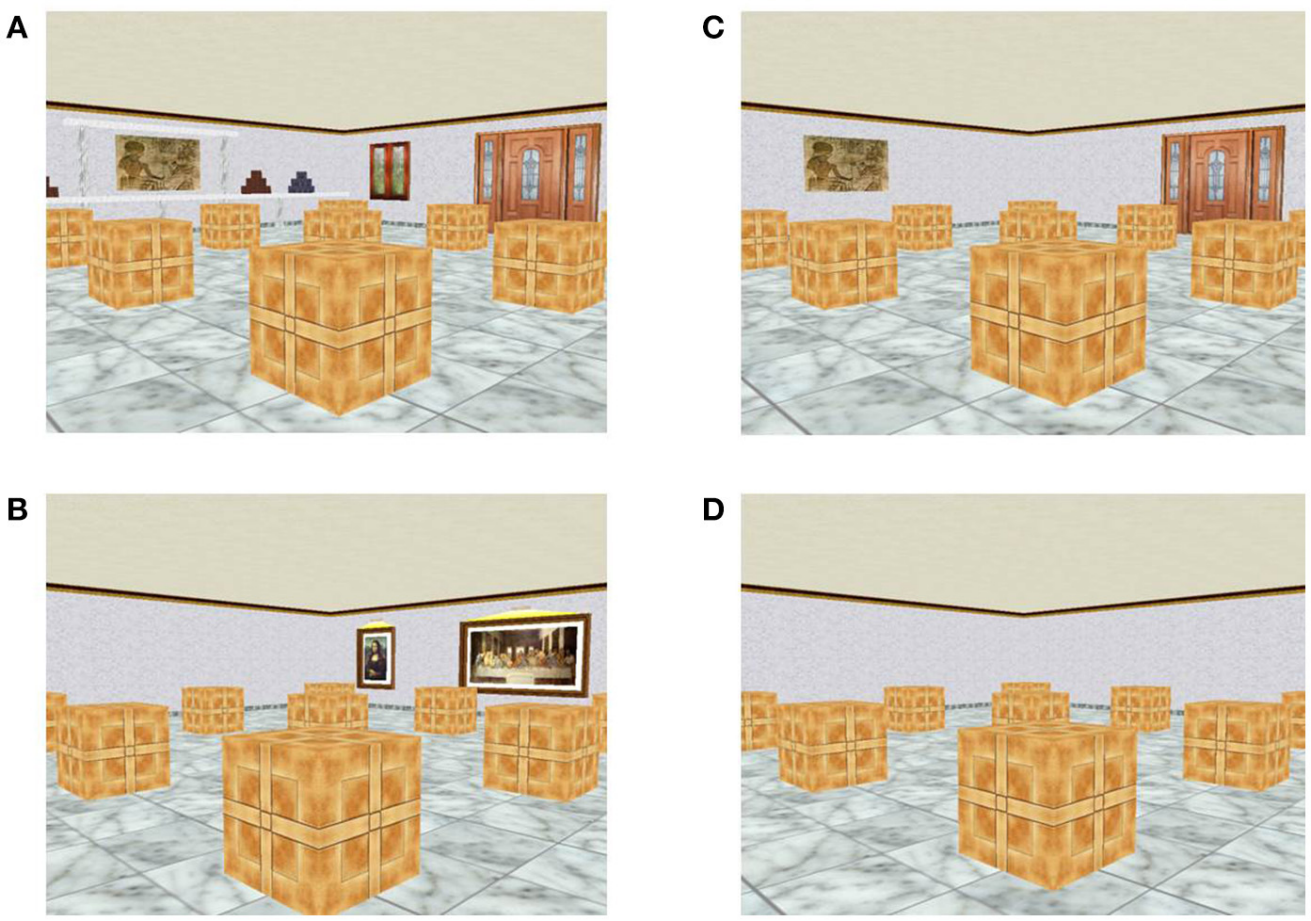
Different conditions applied in the spatial recognition task. **(A,B)** Different perspectives of the room with all landmarks available (AL condition). **(C,D)** Different perspectives of the room with only two landmarks available (2L condition). Note that two walls did not contain any landmark.

Based on the fact that FI and FD individuals perform differently in complex and simple environments, two contextual conditions were administered. In the first one, all possible landmarks (AL) were available in the virtual room represented in the picture (see Figures 2A,B). Every room wall but one had one or more cues. In the two landmarks condition (2L) a door and a picture were available in adjacent walls (see Figures 2C,D).

### Statistical analyses

The median (33.06) of the averaged times on the Embedded Figures Test (in seconds) was used to divide the group into FI (i.e., higher times than median) and FD (i.e., lower times than median) groups. A chi-square was used to determine if the proportion of men and women changed in FD and FI groups.

The number of correct answers and time spent in every condition (AL and 2L) were analysed using a two-way ANOVA (Gender x CS). Tukey test was applied for post-hoc analyses and differences were considered statistically significant for p < 0.05. STATISTICA 10 was used to run analyses.

## Results

A chi-square test showed that proportion of men and women did not differ in FI and FD groups, X^2^ = 0.4, *p* = 0.527.

### All landmarks condition (AL)

#### Accuracy (number of correct answers)

ANOVA (Gender × CS) did not reveal any significant main effect of Gender *F*_(1, 36)_ = 0.001, *p* = 0.977, η_*p*_^2^ = 0, CS *F*_(1, 36)_ = 2.594, *p* = 0.116, η_*p*_^2^ = 0.06, nor Gender × CS interaction *F*_(1, 36)_ = 0.065, *p* = 0.801, η_*p*_^2^ = 0.002.

#### Latency

**Figure 3:**
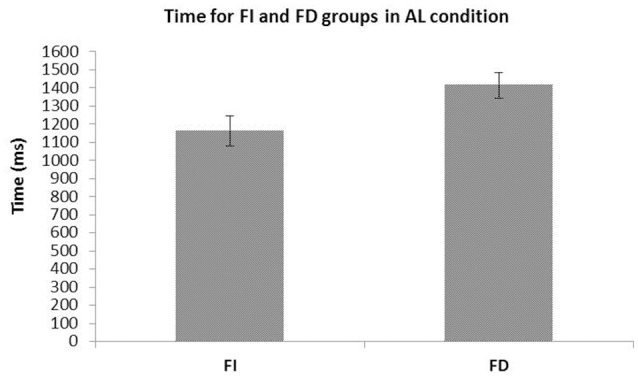
Mean and S.E.M. of time by Field Independent (FI) and Field Dependent (FD) groups to perform the recognition task with all landmarks available. The FI group was faster than the FD one.

The time to perform the recognition task was analyzed with ANOVA (Gender × CS) and revealed a significant main effect of CS *F*_(1, 36)_ = 5.265, *p* = 0.02, η_*p*_^2^ = 0.13. No differences emerged neither in Gender factor *F*_(1, 36)_ = 0.022, *p* = 0.88, η_*p*_^2^ = 0.001, nor Gender × CS interaction *F*_(1, 36)_ = 0.141, *p* = 0.709, η_*p*_^2^ = 0.004. FI group response was faster in the recognition task (1162.7 vs. 1413 ms for FI and FD groups, respectively) (see Figure 3).

### Two landmarks condition (2L)

#### Accuracy (number of correct answers)

**Figure 4:**
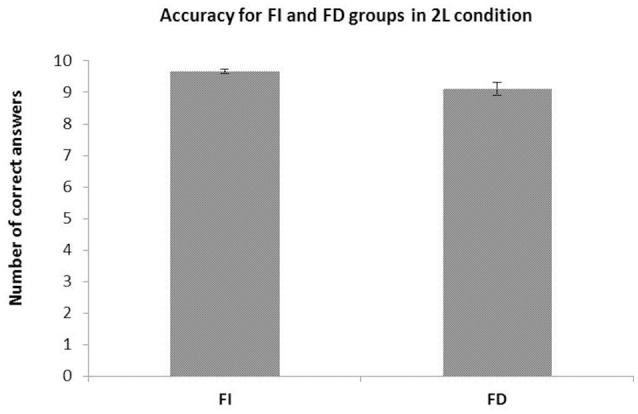
Mean and S.E.M. of number of correct answers for Field Independent (FI) and Dependent (FD) groups in the recognition task with only two available landmarks. The FI group showed a higher accuracy.

ANOVA (Gender x CS) disclosed a significant main effect of CS *F*_(1, 36)_ = 6.505, *p* = 0.015, η_*p*_^2^ = 0.15. No significant main effect was found either in Gender factor *F*_(1, 36)_ = 0.027, *p* = 0.869, η_*p*_^2^ = 0.001 or in Gender × CS interaction *F*_(1, 36)_ = 0.777, *p* = 0.383, η_*p*_^2^ = 0.02. FI participants obtained a higher number of correct answers than those in the FD group (9.66 vs. 9.11 correct answers for FI and FD groups, respectively) (see Figure 4).

#### Latency

ANOVA (Gender x CS) did not reveal any effect of Gender *F*_(1, 36)_ = 0.295, *p* = 0.591, η_*p*_^2^ = 0.008, CS *F*_(1, 36)_ = 2.626, *p* = 0.114, η_*p*_^2^ = 0.06 nor Gender × CS interaction *F*_(1, 36)_ = 0.017, *p* = 0.896, η_*p*_^2^ = 0.0.

For means and SD see Table 2.

**Table 2:** Mean and SD for gender and cognitive style.

			**Mean**	**SD**
AL	Accuracy	Men	9.5	0.72
		Women	9.46	0.6
		FD	9.65	0.14
		FI	9.31	0.14
	Latency	Men	1,267	383
		Women	1,308	327
		FD	1,413	306
		FI	1,162	357
2L	Accuracy	Men	9.43	0.67
		Women	9.34	0.76
		FD	9.11	0.89
		FI	9.66	0.3
	Latency	Men	1,447	455
		Women	1,398	348
		FD	1,523	288
		FI	1,323	475

## Discussion

Relationship between CS and spatial memory performance was assessed in this study. Analyses revealed that FI participants were more accurate than FD when few landmarks were available in the environment (2L) and they were faster than FD when all landmarks were available (AL). Note that in both conditions the virtual room was the same although the only significant change was to be found in the number of cues available.

Taking into account that FI outperform FD in tasks where spatial information needs to be cognitively handled (Witkin, 1977), such as mental object rotation, perspective taking and non-rotating maps usage (Boccia et al., 2016; Li et al., 2016), it is not unusual that they made fewer errors than FD in the 2L condition. FI individuals are more capable than FD in extracting prominent information from the environment and putting them in other's perspective to imagine what they are looking at Witkin (1977). The capacity to extract the prominent information is named disembedding and the ability to imagine other's perspective is known as perspectivism (Witkin, 1977) and both are essential for performing the spatial memory recognition task used in this study. Hence, participants needed to remember one picture and interpret locations from other viewpoints.

In the complex environment, once more we found that FI outperformed FD participants, although group differences emerged on latencies. It is therefore likely that FD individuals could not extract important spatial information and take advantage of it. These findings are in line with subjects' spatial style, where “landmark style” participants are prone to analyse useless details of the environment making difficult their spatial orientation (Siegel and White, 1975; Piccardi et al., 2016). Piccardi et al. (2016) found that “landmark style” subjects were able to recall familiar landmarks but they did not relate them with spatial information. In this regard, they proved to be poor navigators. The eye movement pattern of “landmark style” individuals is characterised by a greater number of fixations of short duration focusing their exploration on the path and related targets. However, survey style individuals explored the environment more comprehensively, focusing their attention on salient cues (Piccardi et al., 2016). In the current research, FD participants may have analysed useless spatial information and, even in the absence of different accuracy, the time required to complete the task was higher for FD than FI. Having excessive information available could affect the FD individuals' performance, since they do not rely on important spatial information. They may spend more time because they use external reference frames which are not reliable for identifying cues in unknown settings (Witkin, 1977).

Regarding gender, Siegel and White (1975) have noted that there are different strategies to solve spatial tasks depending on the information chosen to navigate. These strategies are named “spatial styles.” Some studies have found that women are usually prone to use “landmark” or “route styles,” where egocentric information is necessary. On the contrary, men usually choose a “survey style,” which implies allocentric information and capacity to represent the environment as a map (Coluccia and Louse, 2004; Nori et al., 2006; Nori and Piccardi, 2015). This is evident in tasks where they have to indicate how to reach a target: men normally provide cardinal points and distance information whereas women prefer to add information about landmarks (Miller and Santoni, 1986; Ward et al., 1986; Schmitz, 1997; Dabbs et al., 1998; Denis et al., 1999). This supports that FD and FI may be related to “landmark” and “survey styles,” respectively (Boccia et al., 2017). “Route style” individuals could be both FD and FI since both men and women use this type of orientation (e.g., Boccia et al., 2017).

No gender differences have been revealed in our study. It is well known that dimorphism requires an optimum level of difficulty. When the level of difficulty is low both genders performed accurately. Conversely, very high difficulty levels produce an increase of errors which also increase dispersion and reduce the possibility of finding group differences (Cánovas et al., 2008; León et al., 2014; Tascón et al., 2017). In this study we have shown that men and women displayed a similar performance. We presume that increasing difficulty would disclose a differential performance in both genders, as revealed by Tascón et al., (León et al., 2014) in similar tasks with higher difficulty levels. Another possible interpretation may be related to the distribution of men and women in the FD and FI groups, even if literature shows that women are generally more FD than men, in our sample men and women are equally distributed in the two groups. Boccia et al. (2017) demonstrated that women and men with the same CS did not differ in their performance in spatial memory tasks, adopting similar strategies. Moreover, when both gender adopted the same strategies i.e., military pilots with high spatial abilities, gender differences never appear (Verde et al., 2013, 2015, 2016).

The sample could be a limitation of this study. Although the sample was not too small according to the number of participants included in other studies using the same task (Tascón et al., 2016, 2017), when assessing cognitive styles samples are slightly bigger (Boccia et al., 2016, 2017). A bigger group would allow limiting FI and FD groups to those individuals with extreme values.

As a conclusion, it is important to highlight that different spatial contexts can modify the performance in spatial tasks depending on the CS assumed. FD participants use landmark information to navigate, so a very complex environment makes them not to focus on essential spatial information like landmarks relationship that would facilitate task resolution. FI individuals rely on internal frame references that make them better navigators.

## Author contributions

LT, MB, LP, and JC: Conception and design, data collection, interpretation, critical revision, and manuscript preparation.

### Conflict of interest statement

The authors declare that the research was conducted in the absence of any commercial or financial relationships that could be construed as a potential conflict of interest.

## Abbreviations

2L: two landmarks condition
AL: all landmarks condition
CS: cognitive style
FD: field dependence/dependent
FI: field independence/independent.
